# Approaching rice domestication in South Asia: New evidence from Indus settlements in northern India

**DOI:** 10.1016/j.jas.2016.04.018

**Published:** 2017-02

**Authors:** J. Bates, C.A. Petrie, R.N. Singh

**Affiliations:** a Department of Archaeology and Anthropology, University of Cambridge, UK; b Department of AIHC and Archaeology, Banaras Hindu University, India

**Keywords:** Rice (*Oryza sativa*), Indus Civilisation, South Asia, Macrobotanical analysis, Cultivation systems

## Abstract

The nature and timing of rice domestication and the development of rice cultivation in South Asia is much debated. In northern South Asia there is presently a significant gap (*c*.4200 years) between earliest evidence for the exploitation of wild rice (Lahuradewa *c*.6000 BCE) and earliest dated evidence for the utilisation of fully domesticated rice (Mahagara *c*.1800 BCE). The Indus Civilisation (*c.*3000–1500 BCE) developed and declined during the intervening period, and there has been debate about whether rice was adopted and exploited by Indus populations during this ‘gap’. This paper presents new analysis of spikelet bases and weeds collected from three Indus Civilisation settlements in north-west India, which provide insight into the way that rice was exploited. This analysis suggests that starting in the period before the Indus urban phase (Early Harappan) and continuing through the urban (Mature Harappan/Harappan), post-urban (Late Harappan) and on into the post-Indus Painted Grey Ware (PGW) period, there was a progressive increase in the proportion of domesticated-type spikelet bases and a decrease in wild-types. This pattern fits with a model of the slow development of rice exploitation from wild foraging to agriculture involving full cultivation. Importantly, the accompanying weeds show no increased proportions of wetland species during this period. Instead a mix of wetland and dryland species was identified, and although these data are preliminary, they suggest that the development of an independent rice tradition may have been intertwined with the practices of the eastern most Indus peoples. These data also suggest that when fully domesticated *Oryza sativa* ssp. *japonica* was introduced around 2000 BCE, it arrived in an area that was already familiar with domesticated rice cultivation and a range of cultivation techniques.

## Highlights

• We present new data for rice cultivation in the Indus Civilisation.
• These data suggest rice domestication was linked with the east of this civilisation.
• Rice domestication and cultivation need to be disentangled as issues.
• Results suggest complex rice cultivation systems before c.1500 BCE.

## 1 Introduction

**Fig. 1:**
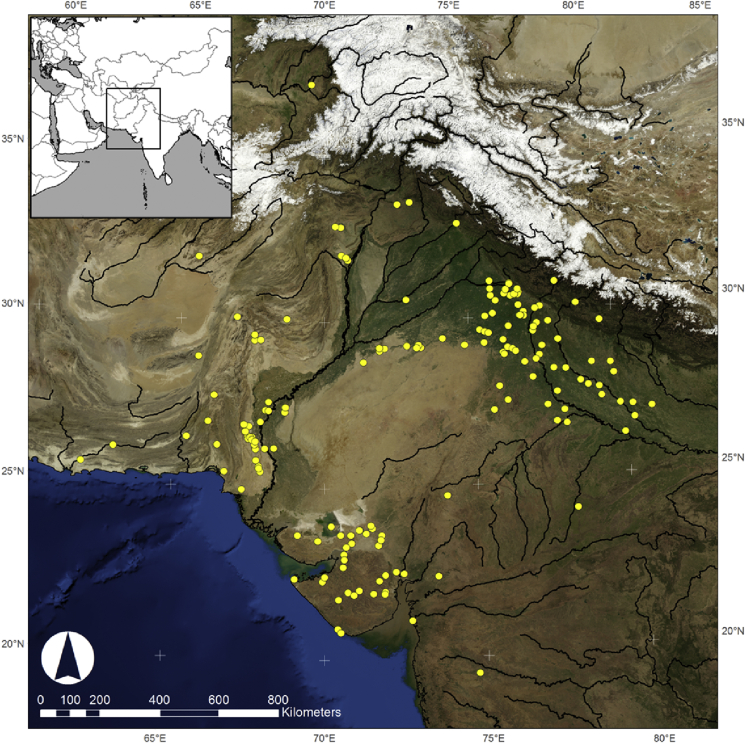
Map showing the distribution of excavated sites belonging to the Indus Civilisation and Painted Grey Ware periods, based on published data as of date of paper submission. Data obtained from in Indian Archaeology, a Review and Possehl (1999).

Since the rediscovery of South Asia's Indus Civilisation (*c.*3000–1500 BCE) (Fig. 1, Table 1) in the early 1900s, the nature of the agricultural practices used by Indus populations has been a source of speculation (e.g. Marshall, 1931; Wheeler, 1953, Fairservis, 1961, Fairservis, 1967). In particular, the role of rice in Indus agriculture has been a continuing source of debate, which is at least partly due to its long and complex history of exploitation in the subcontinent (see Fuller et al., 2010). This paper contributes new evidence to the Indus rice debate by presenting an analysis of archaeobotanical data collected from three settlement sites in the most easterly part of the area occupied by Indus populations. First it will outline the history of rice in South Asia, and it will then review how the Indus Civilisation fits into this debate, before presenting the new evidence and then assessing its significance.

**Table 1:** Periodisation of the Indus Civilisation (after Possehl, 2002:29).

Stage	Dates
Early Harappan	3200–2600 BCE
Early-Mature Harappan Transition	2600–2500 BCE
Mature Harappan	2500–1900 BCE
Late Harappan	1900–1300 BCE
Painted Grey Ware (PGW) (early Iron Age)	1300–500 BCE

## 2 Background

### 2.1 Rice domestication and South Asia

Modern domesticated rice, *Oryza sativa*, has a complex history as it is the product of repeated instances of hybridization. Current genetic evidence suggests that it developed from the hybridization of two other domesticated forms: *O. sativa* ssp. *japonica*, which is a Chinese rice domesticated from wild *O. rufipogon* around 4000 BCE (Fuller and Weisskopf, 2011), and *O. sativa* ssp. *indica*, which is a domesticated version of the South Asian *O. sativa* ssp. *nivara* (Fuller et al., 2010). Based on this evidence, Fuller, 2005, Fuller, 2006, Fuller, 2011 has suggested that *O. sativa* ssp. *indica* may have been domesticated many times, including during what he has referred to as a ‘proto-indica’ phase of cultivation (Fuller, 2011). Using a combination of genetics, the modern distribution of wild rice species, and archaeological evidence, Fuller, 2002, Fuller, 2005, Fuller, 2006, Fuller, 2011, Fuller and Weisskopf, 2011) has also suggested that one of these domestication events may potentially have taken place in the Ganges region of India.

Fuller and Madella (2002) have, however, long argued that the archaeological evidence for rice exploitation in South Asia is patchy and often inconclusive. Based on what is available, Fuller (2011: 82) has proposed that the “independent rice tradition in north India […] never […] proceeded on its own to full domestication” until the arrival of *O. sativa* ssp. *japonica c*.2000 BCE. The earliest evidence for rice cultivation in South Asia comes from the site of Lahuradewa, which is situated in the Middle Ganges plains in north India. Tewari et al. (2008) have recovered charred rice grains from the site that have been radio-carbon dated to 6409 BCE (8359 cal BP) (Tewari et al., 2008: 350), and based on grain length, width and thickness ratios they have suggested that the rice was a domesticated variety. Fuller et al. (2010) have, however, noted that the morphometrics for these grains from Lahuradewa overlap significantly with those of wild grains, and have therefore argued that Lahuradewa could instead represent the beginnings of a long history of cultivation of wild rice that continues throughout the sites occupation. Other sites such as Balu, Banawali and Kunal (Saraswat and Pokharia, 2000, Saraswat and Pokharia, 2002, Saraswat and Pokharia, 2003) provide evidence of rice that is poorly dated but roughly place its use within the third millennium BC (see below) while wild rice was also noted at Senuwar 2 in the Middle Ganges (Saraswat, 2005). Until recently the earliest evidence for domesticated rice based on spikelet base evidence was from the site of Mahagara in the same region, c.1800–1600 BCE. However, as Fuller et al. (2010) have remarked, this attestation is representative of the end of the process of domestication, and is likely to date close to the point when there was a hybridization between *O. sativa* ssp. *indica*/*O. nivara* and *O. sativa* ssp. *japonica*.

The presence of rice at sites like Kunal, Balu, Banawali and Harappa (Saraswat and Pokharia, 2000, Saraswat and Pokharia, 2002, Saraswat and Pokharia, 2003, Weber, 2003) has led scholars to question the role of the Indus Civilisation in the development of rice cultivation systems and even in rice domestication (e.g. Fuller and Madella, 2002, Fuller, 2011). Evidence for rice in northern South Asia in the period between the first exploitation of rice (whether wild or domesticated) at Lahuradewa and the later appearance of clearly domesticated agriculturally grown rice at sites like Mahagara has been eagerly sought, and it has been suggested that Indus Civilisation settlements could provide it (e.g. Fuller, 2002, Fuller, 2006, Fuller, 2011). The next section will explore how these debates have evolved.

### 2.2 Rice exploitation by Indus Civilisation populations

Indus Civilisation populations inhabited the north-west of South Asia between *c*.3000–1500 BCE, and although settlements were primarily distributed in the Indus and Punjab drainage basin, Indus populations also occupied parts of the Kanuma-Ganges doab (Fig. 1), where theoretically they could have come in contact with, and adopted, rice from the Gangetic region (Fuller and Madella, 2002).

Arguments for and against the use of rice by Indus populations began when impressions of rice grains were observed in pottery from Indus settlement sites in Gujarat and Rajasthan (e.g. Ghosh and Lal, 1963, Vishnu-Mittre and Savithri, 1975). Evidence of rice grains has also been recovered from several sites in northwest India (e.g. Early Harappan Kunal, Saraswat and Pokharia, 2003; Early Harappan Balu, Saraswat and Pokharia, 2002; Mature Harappan Banawali), but these attestations have not been securely dated, and the chronology presented in the reports is opaque. Evidence of rice phytoliths from Harappa was presented by Fujiwara et al. (1992) who tentatively dated some of their samples to the Mature Harappan period, confirmed by Madella (2003) in contexts *c*. 2200 BCE, although the only macrobotanical evidence for rice grains from the site places it in the Late Harappan period (Weber, 1997, Weber, 2003). As such Possehl (1999: 246) has argued that there is no evidence for rice cultivation before the Mature Harappan period (i.e. pre-*c*.2500 BCE). Fuller and Madella (2002: 336–7) have argued that “rice was *available* as a crop […] but not adopted” and “there is no reason as yet to believe it was an important crop”, while Fuller and Qin (2009) have argued that there is no evidence of rice agriculture until the Late Harappan period *c*.2000 BCE, when it is likely *O. sativa* ssp. *japonica* arrived. More recently Madella (2014: 230) has considered whether the role of rice changed over time from a secondary crop in the late Mature Harappan to become a staple crop either in the Late Harappan periods or the Early Historic periods. He suggested that rice may have been a secondary but sought after product by Indus Civilisation peoples, explaining its appearance at Harappa, outside its natural habitat and in only small quantities. Madella (2014: 230) also argued that rice only became a staple when its status as a rare crop was lost as superior strains were introduced *c*. 1900BCE, and as diversification in agricultural strategies occurred during the Late Harappan period and into post-Harappan periods.

Three major issues arise from these interpretations. Firstly, there has been a consistent lack of systematic archaeobotanical sampling from Indus sites and many of the rice remains recovered have been of the larger and more obvious grains (Bates, 2015). Secondly, models that differentiate wild gathering, semi-domesticated or wild cultivation, and domesticated agriculture have been developed without an assessment of the spikelet bases at Indus settlements to ascertain how the numerical proportions of wild and domesticated varieties changed over time. Furthermore, the dating of rice use at Indus Civilisation settlements remains problematic (Petrie et al., 2016a).

A lack of systematic archaeobotanical sampling has long bedevilled South Asian archaeology, and the evidence from Indus sites has typically been presented as presence/absence data with little indication of how crop seed grains were recovered. Furthermore, although it has long been argued that grains alone are not suitable for analysis of domestication (Thompson, 1996, Harvey, 2006, Fuller and Weisskopf, 2011), archaeobotanical publications for South Asian sites typically only discuss grains, and neglect to consider weeds and crop processing residues.

There have been several attempts to differentiate wild and domesticated rice in South Asia. Harvey (2006) conducted studies comparing the length: width: thickness ratios of rice reference and archaeological material and concluded that there was too much overlap in the morphometrics of wild and domesticated species, in particular between the wild *O. nivara* and *O. rufipogon*, and between *O. nivara* and its domesticated form, *O. sativa* ssp. *indica*. Recently Castillo et al. (2015) have re-evaluated the use of grain morphometrics to distinguish domestication in rice, and have suggested that some distinction can be made between *O. sativa* ssp. *indica* and *japonica*, but they also note that no distinction can be made between wild and domesticated rice grains using this method. In contrast, spikelet bases have been observed to change morphologically during the domestication process, due to changes in seed dispersal mechanisms (Thompson, 1996). Wild spikelet bases have smooth scars as the rachis shatters to allow for seed dispersal, while domesticated spikelet base scars are rough, because the rachis is non-shattering (Harvey, 2006, Thompson, 1996). Spikelet bases are far smaller than grains, and are often not visible to the naked eye in soil, so they are likely to have been missed at sites where only hand-collecting of remains has been carried out. Analysis of the smaller fractions of floated samples is necessary for gathering such data, but this approach is not often carried out in South Asian excavations (Harvey, 2006).

The complexities of this situation are compounded by the fact that the dating of Indus rice in particular remains vexed. Although rice grains have been noted from the Early and Mature Harappan site of Balu (Saraswat and Pokharia, 2002, Saraswat, 2002), the contexts from which these grains come is unclear, and the date of rice use is difficult to ascertain. For example, the Early and Mature Harappan occupation at Balu has been given the date range of 2300–1700 BCE (Saraswat and Pokharia, 2002, Saraswat, 2002), which spans both the Mature and Late Harappan periods (Petrie et al., 2016a). The presence of rice has also been noted at Kunal (Saraswat and Pokharia, 2003), but the lack of clear contextual information again makes assessing the precise date of its use difficult to ascertain (Petrie et al., 2016a).

In addition to these issues, the date and impact of the shift to wetland rice cropping has also been debated. For example, Coningham (1995: 66–67) has hypothesised that during the post-Indus period there were changes in the methods of growing crops, particularly rice, with a shift from dry to wet land rice. He speculated that with wetland rice exploitation there might have been an increase in yield (kg per acre), which could have supported the rise of even larger urban centres than seen in the preceding Indus Civilisation period (Coningham, 1995: 66–67). This argument was based on the presumed preference for different ecologies of the two main rice crops, as both the wild *nivara* and domesticated *indica* grow in drier conditions than *rufipogon* or *japonica*. However, Fuller and Qin (2009) have noted that all rice species prefer wetter conditions, and can be exploited in a wide range of conditions. They have instead argued that hybridization did not necessarily have to lead to a sudden shift in cropping system towards wetland irrigated rice, and that a more mixed strategy may have been seen, with a range of wet and dry cropping exploited an it is today in some areas of South Asia (Fuller and Qin, 2009). Exploring when wetland rice was introduced and the impact it had is, however, important as wetland systems do increase yield as noted by Coningham (1995). In order to identify this transition, the weed flora must be considered, but it is often not reported in detail in archaeobotanical studies (Fuller and Qin, 2009). In the absence of weed data, Fuller and Qin (2009: 104) relied on the percentage-presence of wet and dry weed taxa from several sites across northern India from the Neolithic to Early Historic periods, and suggested that an increase in the amount of wetland species and a decrease in the presence of dryland species is evident, with only dryland species disappearing over time. However, their study does not take into account the role of the Indus Civilisation in this process. Given the new finds of securely dated rice grains (Petrie et al., 2016a) and the associated spikelet bases reported in this study, the Indus Civilisation becomes an important part of the picture of rice cultivation strategies in the subcontinent.

Our understanding of rice exploitation by Indus populations and the development of rice agriculture during this period in South Asia thus remains patchy and poorly understood, as highlighted by Fuller and Madella in 2002. This paper will attempt to fill some of these gaps and consider how rice exploitation may have developed over time in north-western South Asia. To do this, it will present new archaeobotanical data from settlement sites in northwest India, which lies in the north-east of the Indus region.

## 3 New excavations at Indus settlements on the plains of north-west India

**Fig. 2:**
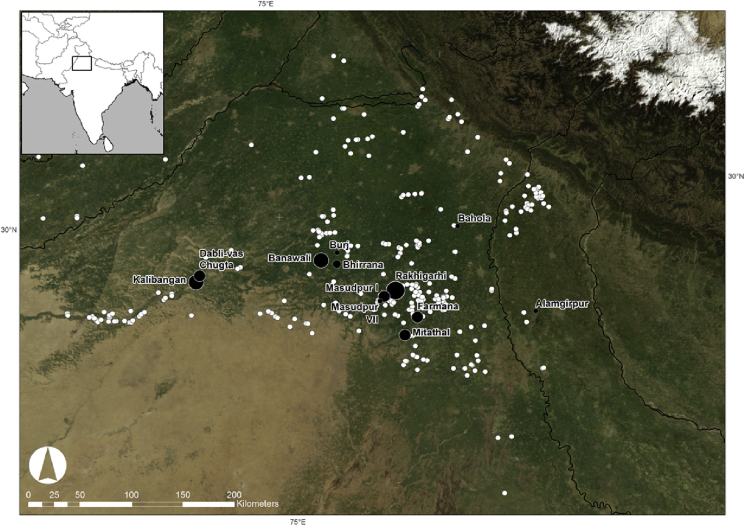
Six sites (Dabli vas Chugta, Burj, Masudpur VII and I, Bahola, and Alamgirpur) excavated by the Land, Water, Settlement Project and their spatial relationship to other Indus sites. (Source: Petrie, pers. com.).

Recent excavations in north-west India by the *Land, Water and Settlement* project have yielded rice grains and spikelet bases from systematically collected flotation samples from three Indus settlements. *Land, Water and Settlement* is a collaborative project between the University of Cambridge and Banaras Hindu University that is operating with the support of the Archaeological Survey of India, and is co-directed by C.A. Petrie and R.N. Singh, and since 2008 the project has conducted surveys and excavated six Indus period village settlements in Rajasthan, Haryana and Uttar Pradesh (Singh et al., 2008, Singh et al., 2010a, Singh et al., 2010b, Singh et al., 2011, Singh et al., 2012a, Singh et al., 2012b, Singh et al., 2013a, Singh et al., 2013b, Petrie et al., 2009, Petrie et al., 2016a, Petrie et al., 2016b; also Pawar, 2012) (Fig. 2). As part of the *Land, Water and Settlement* environmental sampling programme, soil samples were floated using a bucket flotation system and a 500 μm mesh. These samples from three of the sites have produced significant quantities of rice spikelet bases: Masudpur VII (Early-Mature-Late Harappan), Masupdur I (Mature Harappan) and Bahola (Late Harappan-PGW).

Masudpur VII (known locally as Bhimwada Jodha) is a 1-ha “small village” site in Hissar District, Haryana (Petrie et al., 2009: 45), situated within 15 km of the Indus city of Rakhigarhi. Two trenches were excavated – YB2 and YB1 – and a range of local and non-local artefacts were found, including a gold bead and a lapis bead (Petrie et al., 2009). Radiocarbon dating and the associated ceramic material suggested this site was established in the Early Harappan period, occupied during the earlier parts of the Mature Harappan, and also during the Late Harappan period (Petrie et al., 2016a).

Masudpur I (known locally as Sampolia Khera) is a 6-ha “large village” site also in Hissar (Petrie et al., 2009: 39), which is situated approximately 12 km from Rakhigarhi. Three trenches were excavated – XA1, YA3, XM2 – and a wide range of cultural material was found including several beads made of non-local materials like carnelian and faience (Petrie et al., 2009). Radiocarbon dates from the trenches and the associated ceramic material indicate that the site was occupied in the middle and later parts of the Mature Harappan period (Petrie et al., 2016a).

Bahola is a 1–2 ha “small village” site in Karnal district with Late Harappan, PGW and Early Historic occupation (Singh et al., 2013a: 7). One sounding trench – AB1 – and a section cleaning – YK3 – were excavated, but only material from AB1 was collected for flotation. As at Masudpur I and VII, local and non-local artefacts were found including agate and faience objects (Singh et al., 2013a). Radiocarbon dating has not yet been carried out on material from Bahola, but flotation was carried out on soil samples taken from a range of context types.

Rice (*Oryza* sp.), several varieties of millet (*Echinochloa* cf. *colona*, *Setaria* cf. *pumila* and *Panicum* sp.) and a range of tropical (also called *kharif* or summer) pulses (*Vigna mungo*, *Vigna radiata*, *Vigna unguiculata*, *Macrotyloma uniflorum*) were found alongside barley (*Hordeum vulgare*), wheat (*Triticum* sp.) and *rabi* (winter) pulses (*Lens* cf. *culinaris*, *Pisum* sp., *Cicer* sp., *Lathyrus* sp.) at all three sites (Bates, 2015, Petrie et al., 2016a). Rice spikelet bases were also recovered from a range of contexts at both sites (Bates, 2015), including deposits that have been dated to Early Harappan, Mature Harappan, Late Harappan and PGW periods on the basis of relative comparanda (Petrie et al., 2009, Petrie et al., 2016a, Singh et al., 2012a, Singh et al., 2013a). Following the discovery of rice grains at these site, a programme of directly dating rice grains was carried out as part of a wider programme of dating the use of summer crops at Masudpur I and VII (Petrie et al., 2016a). These dates demonstrates that rice was being exploited in both Mature and Late Harappan periods, and the recovery of rice grains and spikelet bases from stratigraphically earlier contexts that were direct dating through dates on other crop species shows that rice was also used as early as the Early Harappan period (Petrie et al., 2016a).

## 4 Analytical methodology

### 4.1 Spikelet bases

**Fig. 3:**
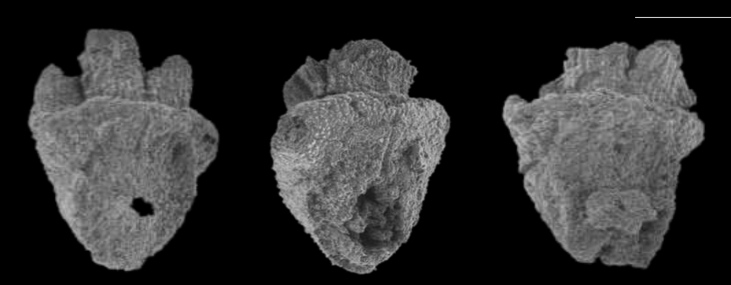
SEM images of rice spikelet bases. (Left) wild type with indented, smooth scar, (Middle) domesticated type with indented ragged scar, (Right) immature type with out-jutting ragged scar. Line at top right shows 500 μm scale. Images J. Bates.

Following their identification, the spikelet bases were separated into wild, domesticated and immature types based on their abscission scars. Following Thompson, 1996, Harvey, 2006 and Fuller and Qin (2009), the criteria for categorising the spikelet bases were as follows (see Fig. 3):

• Wild – shallow circular indented abscission scar with smooth edges and a circular pit
• Domesticated – reniform indented scar with ragged edges and an upstanding stump of tissue or a sub-circular pit
• Immature – out-jutting scar (Fuller and Qin, 2009, Fuller et al., 2010; note that it is important to distinguish between mature wild/domesticated and immature grains as during the process of domestication the proportion of immature rice collected should decrease as grain maturation time narrows and becomes more even across the crop)
• Uncertain – any spikelet bases where the abscission scar had been damaged were categorised as uncertain.

Fuller and Weisskopf (2011) have outlined a simple model for identifying the domestication process of rice, which is applied here. They argued that in a wild rice harvest only wild and immature types will be collected. During periods of cultivation of wild stands, domestication can occur slowly, and the proportion of domestic types increases while the proportion of wild and immature spikelet bases decreases, until finally domesticated types dominate the assemblage, which suggests cultivation of a fully domesticated crop. Fuller and Weisskopf (2011) equated such fully domesticated crops with “agriculture”, and suggest that wild types will persist in a fully domesticated crop as weeds, comprising up to 20% of the spikelet base assemblage (Fuller and Weisskopf, 2011). This model has been applied to Chinese sites (Fuller et al., 2009) and Chinese and Thai rice samples (Fuller et al., 2010), and the authors have argued that no absolute proportions for ‘a wild harvest’ or ‘a domesticated crop’ should be assigned, as the development of any agricultural system is a gradual process, not a series of events.

For the analysis presented here, the data has been assessed for evidence of gradual change over time rather than looking to assign a ‘stage of development’ (*cf*. Fuller and Weisskopf, 2011). Fuller et al. (2009) were able to apply ANOVA tests to assemblages from China to explore the statistical significance of change over time, but the archaeobotanical remains available from the three *Land, Water and Settlement* sites were not as abundant, so this approach has not been attempted here. Instead simple percentages was used to quantitatively compare the sites, following the less complex initial phases of analysis carried out by Fuller et al. (2009).

### 4.2 Weeds

In addition to spikelet bases, Fuller and Qin (2009) have also used weed assemblages to explore how rice was cultivated. Following Fuller and Qin (2009: 104), the ubiquity of wetland and dryland weed species are here compared by period at each site to explore whether the hypothesised shift from dryland cropping to wetland or irrigated cropping could be seen across the Early Harappan to PGW periods. Species have been grouped into wetland/irrigated and dry/upland following Moody (1989), and have been plotted by period for each site where rice grains where found in the macrobotanical samples.

## 5 Results

### 5.1 Spikelet bases

#### 5.1.1 Masupdur VII

A total of 25 contexts from Masudpur VII contained macrobotanical remains: 10 Early Harappan, 12 Mature Harappan and three Late Harappan. *Oryza* sp. grains were found in Early and Late Harappan contexts, and increased in ubiquity and density in the Late Harappan period. Rice was absent macroscopically from the Mature Harappan contexts, but spikelet bases were found in Early, Mature and Late Harappan contexts. As well as rice, a mixture of other summer and winter crops were found, including wheat, barley, small native millets (*Echinochloa colona* and *Setaria* cf. *pumila*) and winter and summer pulses (Bates, 2015, Petrie et al., 2016a).

Spikelet bases were recovered in only three contexts – one Early Harappan, one Mature Harappan and one Late Harappan. The Early Harappan context presented only one spikelet base and was therefore not included in the analysis. The Mature and Late Harappan contexts, however, each had numerous spikelet bases, which were differentiated using the methodology outlined above, and these are shown in Table 2.

**Table 2:** Number of spikelet bases per 20l sediment and as a proportion of spikelet bases from Mature and Late Harappan contexts at Masudpur VII.

Rice spikelet base type	Context 514	Mature Harappan (%)	Context 515	Late Harappan (%)
Wild	135	75.84%	0	0%
Domesticated	17	9.55%	2	28.57%
Immature	26	14.61%	3	42.86%
Uncertain	0	0%	2	28.57%

Converting these densities into percentages (Table 2), it is clear that in the Mature Harappan context, wild types were the most predominant form, comprising *c*.76% of the spikelet bases, whereas in the Late Harappan context wild forms were not present at all. Instead the percentage of domesticated and immature increased compared with the previous period.

#### 5.1.2 Masupdur I

A total of 29 contexts from Masudpur I contained macrobotanical remains, all from the Mature Harappan period (Bates, 2015, Petrie et al., 2016a). Rice grains were found in over half of the contexts, and formed a large proportion of the crop assemblage. Small native millets (*Echinochloa colona*, *Setaria* cf. *pumila*) and barley also appeared with similar frequency and in large proportions as part of a mixture of winter and summer crops.

Spikelet bases were found in nine contexts, though three of these contained only one spikelet each so were not included in the analysis. The contexts examined and the types of spikelet bases identified are shown in Table 3.

**Table 3:** Number of spikelet bases per 20l sediment and as a proportion of spikelet bases from Mature Harappan contexts at Masudpur I.

Rice spikelet base type	Context 310	Context 314	Context 317	Context 319	Context 321	Context 323	Mature Harappan (%)
Wild	0.5	0	1	29.5	4	118	39.46%
Domesticated	0.75	1.5	0.5	23.5	0.5	12	9.99%
Immature	0	0.5	0	4.5	0	12.5	4.51%
Uncertain	1	4	0	19.5	2.5	151.5	46.03%

After converting these densities into an average percentages of the spikelet base assemblage for the Mature Harappan period (Table 3), it is evident that there were proportionately more wild than domesticated types, but there was also a large portion of unidentifiable examples which may have skewed the data.

#### 5.1.3 Bahola

A total of 30 contexts from Bahola contained macrobotanical remains: ten Late Harappan and 20 PGW (Bates, 2015). Rice grains appeared in 50% of Late Harappan contexts and 60% of PGW contexts, and together with *Echinochloa colona* were the most commonly found cereals. Unlike the two Masudpur sites, Bahola displayed a dominance of summer crops, although some winter crops like barley were still present in smaller quantities. Spikelet bases appeared in 13 contexts in total. However, the four PGW contexts contained few spikelet bases so they have been excluded from this analysis, and of the 9 Late Harappan contexts, three contained only one spikelet each and were therefore not included. The data from the remaining six contexts is shown in Table 4.

**Table 4:** Number of spikelet bases per 20l sediment and as a proportion of spikelet bases from Late Harappan contexts at Bahola.

Rice spikelet base type	Context 125	Context 125b	Context 126	Context 131	Context 137	Context 141	Late Harappan (%)
Wild	0	2.22	0	0	0	0	6.92%
Domesticated	1.33	2.22	0	2.67	2.4	2	33.15%
Immature	0	0	0	0	0	0	0%
Uncertain	1.33	11.11	3	1.33	2.4	0	59.93%

After converting these figures into an average for the Late Harappan period (Table 4), it can be seen that while there was a lot of uncertain material, the proportion of domesticated spikelets was greater than those of the wild spikelets, and no immature spikelet bases were identified.

#### 5.1.4 Contrasting the data

**Fig. 4:**
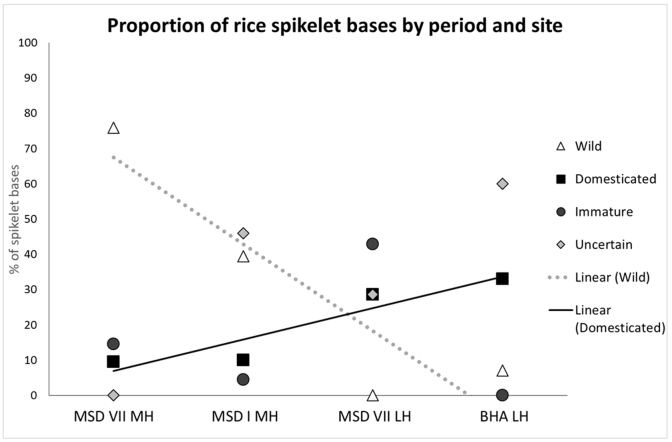
Graph showing the proportion of spikelet base types in chronological order (earliest from left, latest to the right). Lines show the linear regression trendlines. As can be seen, the proportion of domesticated types increased over time and the proportion of wild types decreased over time. Site and period codes have been used: MSD I = Masudpur I, MSD VII = Masudpur VII, BHA = Bahola, MH = Mature Harappan (c.2500–1900BCE), LH = Late Harappan (c.1900–1300BCE).

The average proportions for each site arranged chronologically are shown in Fig. 4 (earliest to the left, latest to the right). Linear regression trendlines are shown, and indicate a strongly correlated negative trend between time and wild spikelet bases (R^2^ value 0.8361) and a strongly correlated positive trend between time and domesticated forms (R^2^ value 0.8758). Comparing this with Fuller and Weisskopf's model (2011), it can be argued that there was indeed a gradual increase in the amount of exploitation of domesticated rice over time. This data potentially provides the first evidence for the ‘proto-indica’ domestication hypothesised for the Gangetic region by Fuller, 2005, Fuller, 2006, Fuller, 2011.

It should also be noted that there is a positive correlation in the uncertain category of spikelet bases with time. This correlation is interesting in association with the positive correlation in domesticated type bases, but whether there is a relationship between the two correlations is difficult to determine. No studies have been carried out to ascertain whether domesticated spikelet bases are more likely to be damaged than other forms, so this positive trend could be coincidental rather than linked with the story of domestication processes. Further research into the breakage patterns of rice spikelet bases could help to untangle these trends and determine if the uncertain spikelet bases seen in this dataset are more likely to have been domesticated types or if no such assumptions can be made.

### 5.2 Weeds

A total of 11 weed species identified in the archaeological assemblages of Masudpur I, Masudpur VII and Bahola could be considered as possible summer rice weeds and assigned as wet/dry/either water preferences (Bates, 2015; after Moody, 1989). The ubiquities of these weeds by period are shown in Table 5, and include examples from contexts that did not contain rice grains and/or spikelets. Ubiquity is a measure of the frequency of occurrence across a site, presented as the percentage of contexts a species was found in.

**Table 5:** Ubiquity of weed species by site and period, with coding in the right most column to indicate species water preference: W (wet), D (dry) and W/D (Wet or dry). Sites and periods have also been coded for simplicity: MSD I = Masudpur I, MSD VII = Masudpur VII, BHA = Bahola, EH = Early Harappan (3200–2600BCE), MH = Mature Harappan (c.2600–1900BCE), LH = Late Harappan (c.1900–1300BCE), PGW = Painted Grey Ware (c.1300–500BCE).

Weed taxa	MSD VII EH	MSD VII MH	MSD VII LH	MSD I MH	BHA LH	BHA PGW	Wet/Dry
*Eleocharis* sp.	80	16.66	33.33	41.38	57.89	50	**W**
*Scirpus* sp.	10	8.33	66.67	6.9	0	10	**W**
*Rumex* sp.	0	0	0	0	5.26	0	**W**
*Coix lachryma-jobi*	0	0	0	3.45	0	0	**W**
*Echinochloa crus-galli*	0	0	0	17.24	5.26	0	**W**
Polygonaceae	10	0	0	13.79	0	10	**W**
*Chenopodium album*	0	0	0	3.45	15.79	10	**D**
*Trianthema triquetra*	30	0	0	17.24	10.53	0	**D**
*Solanum* sp.	10	0	0	6.9	0	0	**D**
*Eragrostis* sp.	0	0	0	13.79	47.37	50	**D**
*Brachiaria* sp.	0	0	0	17.24	0	0	**D**
*Chryspogon* sp.	10	8.33	0	13.79	57.89	10	**D**
Cyperaceae	100	58.33	100	86.21	100	80	**W/D**

**Fig. 5:**
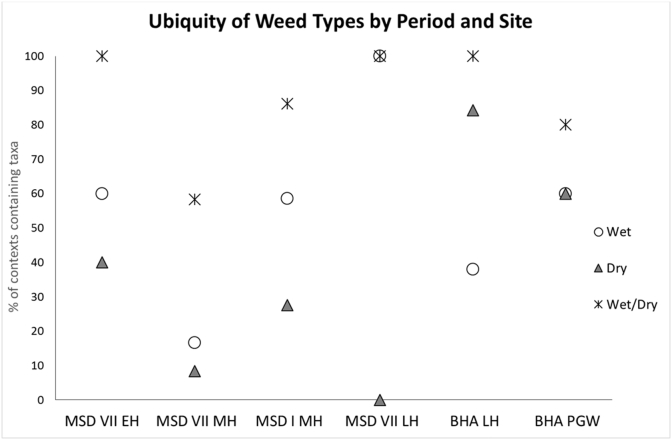
Comparing the ubiquity (% of samples containing the taxa) of wet, dry and wet/dry weeds by period (earliest to the left, latest to the right). Very little by way of patterning can be seen in this data set. There is no clear trajectory of change over time. Sites and periods have been coded: MSD I = Masudpur I, MSD VII = Masudpur VII, BHA = Bahola, EH = Early Harappan (3200–2600 BCE), MH = Mature Harappan (c.2600–1900 BCE), LH = Late Harappan (c.1900–1300 BCE), PGW = Painted Grey Ware (c.1300–500 BCE).

The data from all phases at all sites to show the ubiquity of dry versus wet and wet/dry types is illustrated in Fig. 5.

The data presented here shows that there was no strong positive correlation in wetland species and negative correlation in dryland species. Instead, weak positive correlations are seen in both (R^2^ linear regression values of 0.0411 for wet species and 0.2549 for dry). This is contrary to the hypothesis that rice cultivation would have relied on dryland techniques until the introduction of *O. sativa* ssp. *japonica c*.2000 BCE when wetland techniques would have been required (cf. Fuller and Weisskopf, 2011, Fuller and Qin, 2009), Indeed, the positive correlation for dryland species was slightly stronger than that for wetland species.

Significantly, at Masudpur I and VII there were more wetland weed species than dryland in all periods. In contrast, at Bahola there was a patterning similar to that expected by Fuller and Weisskopf's (2011) hypothesis, as there was a decrease in the ubiquity of dry species and an increase in wet species in the PGW. However, in light of the overall patterns from all three sites it can be argued that the weeds do not fit with the idea of a change towards wetland cropping over time and no sudden shift to wetland species is seen *c*.2000 BCE.

The presence of wet environment weeds does not, however, suggest that complex paddy systems were being used pre-2000 BCE. It is possible that marginal wet-dry environments could have been exploited, or simple irrigation techniques like garbarbands might have been used to trap water seasonally rather than permanently. It is important to remember that the plains of north-west India were clearly within the zone affected by and benefitting from the Indian Summer Monsoon (Dixit et al., 2014, Petrie et al., 2016b).

## 6 Implications of these data

There has been a tendency in archaeology to conflate domestication with agricultural strategies (Harris, 2007), and this is seen in the models of South Asian rice exploitation that have been developed. Harris (2007) has argued that cultivation is any act that promotes plant growth, and can lead to domestication without full agriculture, which he defines as tillage of the land to promote plant growth. The new data from north-western India presented here fills some of the gap between wild ‘cultivation’ at Lahuradewa and domesticated ‘agriculture’ at Senuwar 2 and Mahagara, and suggests that the process of domestication was well underway in northern South Asia before the arrival of *O. sativa* ssp. *japonica* and the form of wet rice agriculture it required. These new data suggests that there may have been the exploitation of domesticated rice before the arrival of wetland rice agriculture, and that rice cultivation needs to be considered as a central issue in discussions of the exploitation of domesticated rice in northern South Asia. We suggest that the debates over rice in South Asia need to be separated into two issues in future analyses, specifically the untangling of the complex issue of the domestication of *O. nivara* to *O. sativa* ssp. *indica* in northern South Asia from the issues related to the development of rice agriculture.

## 7 Conclusions

The evidence for rice grains, spikelet bases and weed species from the three *Land, Water and Settlement* project sites reviewed here illuminates the process of rice domestication in northern South Asia in the period between the wild cultivation seen at Lahuradewa and the evidence of full agriculture from Mahagara. At all three *Land, Water and Settlement* sites there is a pattern of increasing proportions of domesticated and corresponding decrease in wild spikelet types over time. The material from the *Land, Water and Settlement* excavations also demonstrates that the exploitation of rice by Indus populations appears to pre-date the arrival of *O. sativa* ssp. *japonica* and wet rice farming. Furthermore, the weeds suggest that rather than a shift towards wetland cropping during the Late Harappan or PGW periods, as has been previously hypothesised, a complex pattern of exploiting both wet and dry land species is seen in the Early, Mature and Late Harappan periods and also in the post-Harappan PGW phase at these settlements. The analyses of the rice grains, spikelet bases and weeds suggest therefore that the relationship between agricultural strategy and domestication is more complex than has been previously suggested and that rice domestication without paddy fields may have occurred in northern South Asia between *c*.6000 BCE (Lahuradewa) and the arrival of Chinese rice *c*.2000 BCE.

These new data thus demonstrate that rice cultivation has a complicated history in the subcontinent, and needs further consideration with relation both to the nature of Indus agriculture in the region and also to the domestication of rice in northern South Asia. More research incorporating systematic flotation at Indus settlements and also those contemporaneous to the Indus Civilisation is needed to explore the range of cultivation practices being exploited in this complex agricultural and environmental region.
